# Stressors and Coping Strategies during Clinical Practice among Diploma Nursing Students

**DOI:** 10.21315/mjms2019.26.2.10

**Published:** 2019-04-30

**Authors:** Rusnani Ab Latif, Mohd Zarawi Mat Nor

**Affiliations:** 1 Kubang Kerian Nursing College, 16150 Kubang Kerian, Kelantan, Malaysia; 2 School of Medical Sciences, Health Campus, Universiti Sains Malaysia, 16150 Kubang Kerian, Kelantan, Malaysia

**Keywords:** stressors, coping strategies, clinical practice, nursing students, perceived stress scale

## Abstract

**Introduction:**

Consistent with the significant of the stress issue in education, this study aimed to survey type of stressors and identifies the coping strategies used by diploma nursing students during clinical practices.

**Methods:**

A descriptive cross-sectional study was carried out at the Kubang Kerian Nursing College, Kelantan which involved 346 respondents using simple random sampling method. The inclusion criteria were year one, two and three of nursing students who have clinical posting and voluntarily joining the study. Perceived Stress Scale (PSS) and Brief COPE inventory were utilised in the data collection. Higher mean score indicates higher degree of stress.

**Results:**

Clinical assignments and workload were the main stressor (mean = 3.19, SD = 1.09). Religion approach was the most coping strategy applied (mean = 3.30, SD = 0.71). Pearson’s correlation coefficient test found that six domains of stressors during clinical practices (taking care of patients; clinical educators/instructors and ward staff; clinical assignments and workload; peers and nursing students from other college; lack of professional knowledge and skills and clinical environment) were statistically significant correlation with coping strategies, where *P*-value < 0.05.

**Conclusion:**

Clinical assignment was the main stressor among nursing students; therefore, successful activities should be promoted to help them in managing clinical assignment and enhancing knowledge in religion.

## Introduction

Stress is a particularly important issue in education because it has the potential to impede learning and performance (1). Empirical research supports the view that nursing students suffer from stress in their clinical practice (2, 3). Without doubt, clinical practice is a crucial component in nursing education. However, students may face many challenges or threats in dynamic and complex clinical environments, such as how to use high-tech medical equipment, how to maintain good relationships with clinical staff and instructors, how to manage sudden changes in a patient’s condition and how to deal with the demands of patients’ relatives (4). When compared to students from other health-related disciplines, nursing students have been reported to experience higher levels of stress and more related physical and psychological symptoms (5, 6). Students who experience psychological distress are more likely to experience depressive symptoms (7, 8).

The term *stress* “has so many different meanings that it is confusing, elusive, and heard so often its meaning is frequently distorted and its implications taken for granted” (9). Situations in the college environment, especially during the academic lives of nursing students, are considered to be one of several areas of stress exposure (10). Stress exists for students in both the academic and the clinical fields of the study programme (11). Research in the field of work stress illustrates a growing belief that coping is a basic and important factor in the relationship between stressors and distress. *Coping strategies* refers to the specific efforts, both behavioural and psychological, that people employ to master, reduce, tolerate or minimise stressful events.

Sheu et al. (12) identified six stressors during the clinical teaching in nursing (CTN) programme: (i) stress due to patient care, (ii) stress due to tutors and nursing professionals, (iii) stress resulting from tasks and workload, (iv) stress due to partners and daily life, (v)stress due to lack of knowledge and professional competencies and (vi) stress from the clinical context. Coping is a constantly changing cognitive and behavioural effort to manage specific external and/or internal demands that are experienced as taxing or exceeding the resources of the person (11, 12). The top five coping strategies for the undergraduate nursing students in this study were religion, the use of instrumental support, acceptance, active efforts to cope and positive reframing, the majority of which were emotion-focused coping strategies (13). Nevertheless, there is still a need for more research on stressors relating to clinical practice and the use of coping strategies among the Malaysian nursing student population to fill the gap in understanding. Thus, it is important for researchers to explore the use of effective coping strategies in order to help nursing students overcome stress and maintain their health at an optimum level.

Based on the literature, the majority of nursing students face stress during their clinical practice. Thus, we have chosen to explore the stressors and coping strategies during clinical practice among diploma nursing students in the Kubang Kerian Nursing College, Kelantan. It is hoped that the findings from this study will provide faculty and clinical educators/instructors with information to help diploma nursing students manage or overcome the stress associated with clinical practice. Any stressful situations can be overcome by applying appropriate coping strategies. The aim of the study is to identify stress and coping strategies among diploma nursing students during clinical practice in the Kubang Kerian Nursing College, Kelantan.

## Methods

### Research Design

This study is a descriptive cross-sectional study.

### Population and Setting

The study was conducted in the Kubang Kerian Nursing College, Kelantan.

### Sample Size

Sample size was calculated using a single sample mean formula based on the standard deviation of stressor domains (SD = 0.83, with sample size determination, precision = 0.1 and α = 0.05) and coping strategies as reported by a previous study (13). The largest sample size was 283. After a 20% dropout rate, the adjusted sample size was 340 study subjects. The research included approximately half of the students in each semester: 103 first year students, 100 second year students and 143 third year students. Therefore, the study comprised a total of 346 respondents.

### Sampling Method

A simple random sampling technique was used because each individual had the same probability of being selected. The respondents were randomly selected by the researchers from the first to third year nursing student population by taking half the students for each semester as potential respondents.

### Inclusion Criteria

The inclusion criteria for the participants of the study were as follows: year one, two and three diploma nursing students in the Kubang Kerian Nursing College, Kelantan who had experienced a clinical posting. The nursing students who provided informed consent, volunteered to participate and were present at the time of the data collection joined the study.

### Data Collection

The data collection was carried out from 1 January to 30 June 2014.

### Instruments

Questionnaires were used for the data collection process to obtain qualitative data, and the questionnaire comprised three sections:

**Table:** 

Part A:	Socio-demographic factors: age, race, religion, year of study and interest in nursing
Part B:	The Perceived Stress Scale (PSS) questionnaire adapted from Sheu et al. (12)
Part C:	The Brief COPE Inventory adopted from Carver. (14)

Part B consisted of a five-point Likert scale (1 = not stressful at all to 5 = extremely stressful) in order to measure the degree of stress for each of the stressors. The PSS questionnaire was scored after it had been administered to the respondents and a high mean score indicated a high degree of stress.

#### The six stress factors

**Table:** 

Domain I:	Stress from taking care of patients (Q1–Q6).
Domain II:	Stress from clinical educators/instructors and ward staff (Q7–Q13).
Domain III:	Stress from clinical assignments and workload (Q14–Q18).
Domain IV:	Stress from peers and nursing students from other colleges (Q19–Q21).
Domain V:	Stress from lack of professional knowledge and skills (Q22–Q25).
Domain VI:	Stress from the clinical environment (Q26–Q29).

This section consisted of 28 items which measured 14 different coping styles. The items were presented on a four-point Likert scale, in which 1 represented “I usually didn’t do this at all” and 4, “I usually did this a lot.” The Brief COPE Inventory was scored after administration to the respondents. A raw score was calculated for every sub-scale by adding together the values for the items. A higher score on a sub-scale can be interpreted to mean that an individual is prone to use such a coping strategy more frequently.

### Measurement of Variables

The independent variable was the type of stressor and the dependent variable was the coping strategy.

## Validity and Reliability

### Validity

The Perceived Stress Scale (PSS) questionnaire has been validated by Sheu et al. (12). The content validity index of the PSS questionnaire was 0.94, thus proving its validity. In addition, 50.7% of the total variance was accounted for by the six factors, which confirmed the construct validity of the instrument. The Brief COPE Inventory demonstrated moderate validity indices (14, 15).

### Reliability

The alpha coefficients for the 14 coping styles in the Brief COPE Inventory ranged between α = 0.83 and α = 0.92 (14). The test-retest correlations suggested that self-reports of coping tendencies measured by the Brief COPE Inventory were relatively stable (14, 15).

## Data Analysis

All data was analysed using Statistical Package for Social Sciences (SPSS) software version 19.0. The socio-demographic data was analysed using descriptive statistics and was summarised as the mean and standard deviation (SD). Data was analysed using the mean, SD and percentage. The Pearson’s correlation coefficient was used to identify factors associated with the frequency of use of the coping strategies and the significant level was set at α = 0.05.

## Results

### Demographic

A total of 346 respondents participated in this study, yielding a response rate of 100%. Demographic information on the students is presented in Table 1. The majority of the respondents involved in the study were Malaysian (*n* = 328, 94.8%), Indian (*n* = 11, 3.2%) and Chinese (*n* = 4, 1.2%), followed by other nationalities (*n* = 3, 0.9%). The age of the respondents ranged from 18–25 years old. The majority of respondents (*n* = 275: 79.5%) belonged to the 18–21 age group with a smaller (*n* = 71: 20.5%) 22–25 year old age group. With regard to religion, the majority of the respondents were Muslim (*n* = 330, 95.4%), followed by Christian (*n* = 5, 1.4%) and Hindu (*n* = 11, 3.2%). About 130 (37.6%) were from year 1, 137 (39.6%) from year 2 and only 79 (22.8%) from year 3. The number of students interested in nursing was 310 (89.6%) and 36 (10.4%) were not interested in nursing.

### Clinical Stressors Perceived by the Undergraduate Nursing Students

Clinical stressors perceived by the undergraduate nursing students are shown in Table 2. Among the six types of stressors, the most common types of stressors perceived by students were (i) stress from clinical assignments and workload (mean = 3.19, SD = 1.09), followed by (ii) stress from clinical educators/instructors and ward staff (mean = 3.02, SD = 1.18), (iii) stress from the clinical environment (mean = 2.91, SD = 1.13), (iv) stress from peers and nursing students from other colleges (mean = 2.85, SD = 1.05), (v) stress from taking care of patients (mean = 2.52, SD = 1.04) and (vi) stress from lack of professional knowledge and skills (mean = 2.48, SD = 1.07).

### Coping Strategies Frequently Used by Undergraduate Students to Relieve Stress During Clinical Practice

The coping strategies commonly used by nursing students during clinical practice and their effectiveness are presented in Table 3. The most frequently used coping strategy was religion (mean = 3.30, SD = 0.71); for example, ‘I’ve been trying to find comfort in my religion or spiritual beliefs’ or ‘I’ve been praying or meditating’. This was followed by the use of instrumental support (mean = 3.09, SD = 1.79), planning (mean = 2.98, SD = 0.75), positive reframing (mean = 2.90, SD = 0.82), active coping (mean = 2.87, SD = 0.85), acceptance (mean = 2.83, SD = 0.82), use of emotional support (mean = 2.72, SD = 0.91), self-distraction (mean = 2.72, SD = 0.95), venting (mean = 2.41, SD = 0.97), behavioural disengagement (mean = 1.80, SD = 1.32) and substance abuse (mean = 1.18, SD = 0.76).

Table 4 presents the association between domains of stressors and the coping strategies used. Pearson’s correlation coefficient test found that six domains of stressors during clinical practices were statistically significantly correlated with coping strategies, where *P*-value < 0.05. There was a statistically significant correlation between stresses from taking care of patients and the following coping strategies: those where *P*-value < 0.001 were instrumental support (*r* = 0.19, *P* < 0.001), denial (*r* = 0.26, *P* < 0.001), venting (*r* = 0.21, *P* < 0.001), behavioural disengagement (*r* = 0.31, *P* < 0.001) and self-blame (*r* = 0.21, *P* < 0.001). Meanwhile, for the *P*-value < 0.05 were emotional support (*r* = 0.12, *P* = 0.026) and self-distraction (*r* = 0.14, *P* = 0.009).

There was a statistically significant correlation between stress from clinical educators/instructors and ward staff and the following coping strategies: those where *P*-value was < 0.001 were emotional support (0.18, *P* < 0.001); instrumental support (*r* = 0.21, *P* < 0.001), self-distraction (*r* = 0.19, *P* < 0.001), denial (*r* = 0.20, *P* < 0.001), venting (*r* = 0.18, *P* < 0.001), behavioural disengagement (*r* = 0.32, *P* < 0.001).

Meanwhile, those for the *P*-value < 0.05 were active coping (*r* = 0.13, *P* = 0.014), planning (*r* = 0.12, *P* = 0.020), positive reinterpretation/reframing (*r* = 0.13, *P* = 0.011), acceptance (*r* = 0.15, *P* = 0.005) and self-blame (*r* = 0.16, *P* = 0.002).

There was a statistically significant correlation between stress from clinical assignments and workload and the following coping strategies: those where *P*-value was < 0.001 were active coping (*r* = 0.20, *P* < 0.001), religion (*r* = 0.19, *P* < 0.001), self-distraction (*r* = 0.22, *P* < 0.001), denial (*r* = 0.22, *P* < 0.001), venting (*r* = 0.26, *P* < 0.001), behavioural disengagement (*r* = 0.28, *P* < 0.001), instrumental support (*r* = 0.28, *P* < 0.001) and self-blame (*r* = 0.23, *P* < 0.001).

Meanwhile, those with *P*-value < 0.05 were planning (*r* = 0.17, *P* = 0.001), positive reinterpretation/reframing (*r* = 0.18, *P* = 0.001), acceptance (*r* = 0.10, *P* = 0.044) and emotional support (*r* = 0.222, *P* = 0.001).

There was a statistically significant correlation between stress from peers and nursing students from other colleges and the following coping strategies: those where *P*-value was < 0.001 were instrumental support (*r* = 0.20, *P* < 0.001), self-distraction (*r* = 0.18, *P* < 0.001), behavioural disengagement (*r* = 0.22, *P* < 0.001) and self-blame (*r* = 0.19, *P* < 0.001). Meanwhile, those for the *P*-value < 0.05 were positive reinterpretation/reframing (*r* = 0.11, *P* = 0.036), acceptance (*r* = 0.11, *P* = 0.039, emotional support (*r* = 0.13, *P* = 0.012), denial (*r* = 0.15, *P* = 0.004) and venting (*r* = 0.16, *P* = 0.003).

There was a statistically significant correlation between stress from lack of professional knowledge and skills and ward staff and the following coping strategies: those where *P*-value was < 0.001 were instrumental support (*r* = 0.22, *P* < 0.001), self-distraction (*r* = 0.18, *P* < 0.001), denial (*r* = 0.26, *P* < 0.001), venting (*r* = 0.20, *P* < 0.001), behavioural disengagement (*r* = 0.33, *P* < 0.001) and self-blame (*r* = 0.22, *P* < 0.001). Meanwhile, those for the *P*-value < 0.05 were active coping (*r* = 0.12, *P* = 0.018), planning (*r* = 0.12, *P* = 0.022), positive reinterpretation/reframing (*r* = 0.17, *P* = 0.001), acceptance (*r* = 0.15, *P* = 0.005), religion (*r* = 0.15, *P* = 0.003) and emotional support (*r* = 0.17, *P* = 0.001).

Results also showed that there was a statistically significant correlation between stress from the clinical environment and ward staff and the following coping strategies: those where *P*-value < 0.001 were instrumental support (*r* = 0.21, *P* < 0.001), denial (*r* = 0.29, *P* < 0.001), venting (*r* = 0.19, *P* < 0.001) and behavioural disengagement (*r* = 0.36, *P* < 0.001). Meanwhile, those for *P*-value < 0.05 were acceptance (*r* = 0.10, *P* = 0.047), emotional support (*r* = 0.15, *P* = 0.004), substance use (*r* = 0.13, *P* = 0.014) and self-blame (*r* = 0.16, *P* = 0.002).

## Discussion

### Level of Stress and Types of Stressors

Stress in nursing students is an area of growing concern and it may result in psychological distress, physical complaints, behavioural problems and poor academic performance. In this study, stress from clinical assignments and workload was the greatest stress experienced by nursing students. This finding is similar to Shipton (16), who reported that the significant amount of time students spent on writing assignments was described as stressful. One student in the study conducted by Shipton (16) reported that “Care planning in general has been very stressful for me. It takes me forever to write up care plans. It is good, but it takes too much time.” Another student stated: “Just having too much to do. I don’t have adequate time to get it done. A lot of the time, I don’t have time to do anything for myself. No “me time’.”

The findings of a study carried out by Mahat (2) showed that nursing students also perceived heavy workload as one of their clinical stressors. However, more recent studies (Brown and Edelmann) (17), in which supernumerary status was incorporated into the education of nursing students, found that increased responsibility does not produce stress (Evans and Kelly) (18). In clinical practice, stressors among nursing students during clinical practice were significantly correlated with the care of the terminally ill, time pressure for certain activities, clinical trial evaluations, performance evaluations and frequent changes of services/health institutes (12, 19, 20). Students frequently identified the preparation for clinical assignments as stressful. An area of particular concern was the writing of care plans.

### Commonly Used Coping Strategies

Coping with stress, for a student nurse, is a dynamic and ongoing process, aimed at survival, growth and maintenance of individual integrity. In this study, the most frequently used coping strategy was religion, followed by the use of instrumental support, planning, positive reframing, active coping, acceptance, the use of emotional support, self-distraction, venting, self-blame, humour, denial, behavioural disengagement and substance abuse. According to World Health Organization (WHO)/European Hematology Association (EHA) guidelines, there are no standards for coping strategies; instead, they depend on socio-economic factors. In this study, the majority of student nurses tend to use more healthy coping strategies than negative or unhealthy ones. However, this finding was inconsistent with the finding of Hsiao et al. (21), who reported that the most frequently used coping strategies among nursing students at Chiang Mai University were seeking social support (62.25%), systematic problem solving (23.73%) and accepting responsibility (8.47%).

### Research Limitations

The cross-sectional design of this study provided information about the intensity of stress at only a single point in time. Future studies should incorporate a longitudinal design and use a larger and more representative sample to improve generalisability.

## Conclusion

Nursing students are valuable human resources. Detection of potential depression among nursing students is crucial since depression can lead to low productivity, minimised quality of life and suicidal tendencies. Stress has become a chronic and pervasive condition in the world today. Every person experiences different forms of stress throughout their life, and a student nurse is no exception because he or she has to adjust to an entirely new environment upon joining a training course in nursing. In this study, the most common stressor was stress from clinical assignments and workload. Among the fourteen types of coping strategies, religion was the most frequently used. The results provided valuable information for nurse educators and clinical staff in identifying students’ needs, facilitating their learning in the clinical setting and developing effective interventions to reduce the stress they encounter. Nursing is a stressful programme of study. Implementing stress-management techniques in a nursing programme has a positive effect on retention and performance. It has been shown that nursing students perform with less anxiety when using stress-management techniques such as massage. Increasing nurses’ awareness of complementary techniques supports their retention in academic and professional fields.

## Recommendations

Nurse educators and curriculum planners should make a positive contribution toward minimising the stress of student nurses. All nursing clinicians and nurse educators should adequately prepare students before clinical practice and provide them with orientation to the hospital environment and the staff and polices of the clinical training location. All personnel involved in teaching nursing students, including clinicians, need to be adequately prepared to deal with students and be aware of their own impact on students (19). Nurse educators must ensure the effectiveness of a range of support services for students throughout programmes by offering appropriate academic assistance and guidance. The nursing curriculum should be proactive in equipping student nurses with effective coping skills, which can later be called upon in their future nursing careers. It is important to implement teaching strategies whereby student nurses are empowered to gain positive intrapersonal and interpersonal skills and retain their personal identity and self-awareness.

Lastly, further research is needed to assess the coping strategies used by student nurses and assess their effectiveness in reducing stressors. A further study is also needed to compare the stressors and the counselling needs of nursing students across public and private nursing colleges.

## Figures and Tables

**Table 1:** Participant demographics (*N* = 346)

Characteristics	*n*	%
Race		
Malaysian	328	94.8
Indian	11	3.2
Chinese	4	1.2
Others	3	0.9
Age (years)		
18–21 years	275	79.5
22–25 years	71	20.5
Religion		
Muslim	330	95.4
Christian	5	1.4
Hindu	11	3.2
Year of study		
Year 1	130	37.6
Year 2	137	39.6
Year 3	79	22.8
Interest in nursing		
Yes	310	89.6
No	36	10.4

**Table 2:** Stressors perceived by nursing students (*N* = 346)

Stressors	Rank	Mean	SD
**1. Stress from clinical assignments and workload**	1		
I worry about low marks/poor grades.		3.19	1.09
I feel that my performance does not meet clinical educators/instructors’ expectations.		2.76	1.02
I feel that the requirements of clinical practice exceed my ability.		2.31	1.03
I face an excessive number of clinical assignments.		2.46	1.02
The date for clinical assignment submission is too soon (not given enough time to complete the assignments).		2.68	1.11
**2. Stress from clinical educators/instructors and ward staff**	2		
I do not know how to explain/discuss patients’ illness with clinical educators/instructors, medical and nursing personnel.		2.42	0.94
Ward staff like to pass their responsibilities on to nursing students.		3.02	1.18
Clinical instructors/educators scold/commend me in front of the patients.		2.97	1.21
Medical personnel lack empathy and are not willing to help.		2.69	1.12
Ward staff underestimate my ability to perform clinical tasks.		2.52	0.99
There is a lack of care and guidance from clinical instructors/educators and ward staff.		2.40	1.03
I feel that clinical educators/instructors are unfair in their evaluation of students.		2.35	1.09
**3. Stress from the clinical environment**	3		
I feel stressed with the hospital environment where clinical practice takes place.		2.10	0.98
I am unfamiliar with the ward facilities.		2.08	0.96
I feel stressed due to rapid/sudden changes in a patient’s condition.		2.59	1.05
I feel stress due to a gap between theory in lectures and real situations in the clinical practice.		2.91	1.13
**4. Stress from peers and nursing students from other colleges**	4		
I fear the possibility of making an error (e.g. error medication or assessment of patient).		2.85	1.05
I experience competition from peers in same group and nursing students from other colleges.		2.53	1.06
I cannot get along with the peers in the same group.		2.32	1.12
I feel pressure from clinical educators/instructors who evaluate students’ performance by comparison.		2.40	1.08
**5. Stress from taking care of patients**	5		
Lack of experience and ability in providing holistic nursing care and in making judgments.		2.37	0.86
Condition of patients (dying, chronic illness, contagious disease etc.).		2.40	1.00
Worry about not being trusted or accepted by patients and/or patients’ family.		2.430	0.96
Poor communication with the patients.		2.09	1.00
Evaluation of my performance by patient(s).		2.24	0.97
Facing difficulty in changing from the role of a student to that of a nurse.		2.52	1.04
**6. Stress from lack of professional knowledge and skills**	6		
Unfamiliar with professional nursing skills.		2.48	1.07
Unfamiliar with medical history and terms.		2.48	1.01
Unfamiliar with patients’ diagnoses and treatments.		2.34	0.93

**Table 3:** Mean and SD of coping strategies used by undergraduate students (*N* = 346)

Coping strategies/Item	Strategy ranking	Item ranking	Mean	SD
Self distraction	8	8		
1. I’ve been turning to work or other activities to take my mind off things.			2.25	0.82
19. I’ve been doing something to think about it less, such as going to movies, watching TV, reading, daydreaming, sleeping or shopping.			2.72	0.95
Active coping	4	5		
2. I’ve been concentrating my efforts on doing something about the situation I’m in.			2.74	0.81
7. I’ve been taking action to try to make the situation better.			2.87	0.85
Denial	13	12		
3. I’ve been saying to myself: “This isn’t real.”			1.52	0.72
8. I’ve been refusing to believe that it has happened			1.82	0.81
Substance abuse	14	14		
4. I’ve been using alcohol or other drugs to make myself feel better.			1.18	0.76
11. I’ve been using alcohol or other drugs to help me get through it.			1.26	0.89
Use of emotional support	6	7		
5. I’ve been getting emotional support from others.			2.72	0.91
15. I’ve been getting comfort and understanding from someone.			2.68	1.42
Use of instrumental support	2	2		
10. I’ve been getting help and advice from other people.			2.82	0.88
23. I’ve been trying to get advice or help from other people about what to do.			3.09	1.79
Behavioural disengagement	12	13		
6. I’ve been giving up trying to deal with it.			1.80	1.32
16. I’ve been giving up the attempt to cope			1.72	0.75
Venting	9	9		
9. I’ve been saying things to let my unpleasant feelings escape.			2.41	0.97
21. I’ve been expressing my negative feelings.			2.39	0.91
Positive Reframing	5	4		
12. I’ve been trying to see it in a different light, to make it seem more positive.			2.90	0.82
17. I’ve been looking for something good in what is happening.			2.86	0.81
Planning	7	3		
14. I’ve been trying to come up with a strategy about what to do.			2.73	0.74
25. I’ve been thinking hard about what steps to take.			2.98	0.75
Humour	11	11		
18. I’ve been making jokes about it.			1.84	0.82
28. I’ve been making fun of the situation.			1.37	0.63
Acceptance	3	6		
20. I’ve been accepting the reality of the fact that it has happened.			2.76	0.84
24. I’ve been learning to live with it.			2.83	0.82
Religion	1	1		
22. I’ve been trying to find comfort in my religion or spiritual beliefs.			3.30	0.81
27. I’ve been praying or meditating.			3.30	0.81
Self-Blame	10	10		
13. I’ve been criticising myself.			2.34	0.89
26. I’ve been blaming myself for things that happened.			2.21	0.87

**Table 4:** Association between domains of stressors and the coping strategies used

Dependent variables	Domains of stressors											

	Taking care of patients		Clinical educators/instructors and ward staff		Clinical assignments and workload		Peers and nursing students from other colleges		Lack of professional knowledge and skills		Clinical environment	
					
	*r*	*P*	*r*	*P*	*r*	*P*	*r*	*P*	*r*	*P*	*r*	*p*
Active coping	0.14	0.790	0.13	0.014*	0.20	< 0.001*	0.05	0.279	0.12	0.018*	0.05	0.299
Planning	0.02	0.622	0.12	0.020*	0.17	0.001*	0.09	0.069	0.12	0.022*	0.07	0.177
Positive reinterpretation/reframing	0.98	0.69	0.13	0.011*	0.18	0.001*	0.11	0.036*	0.17	0.001*	0.06	0.227
Acceptance	0.80	0.137	0.15	0.005*	0.10	0.044*	0.11	0.039*	0.15	0.005*	0.10	0.047*
Humour	0.06	0.204	0.09	0.070	0.08	0.113	0.06	0.260	0.06	0.285	0.99	0.065
Religion	0.58	0.282	0.10	0.060	0.19	< 0.001*	0.05	0.284	0.15	0.003*	0.08	0.130
Emotional support	0.12	0.026*	0.18	< 0.001*	0.22	0.001*	0.13	0.012*	0.18	0.001*	0.15	0.004*
Instrumental support	0.19	< 0.001*	0.21	< 0.001*	0.28	< 0.001*	0.20	< 0.001*	0.22	< 0.001*	0.21	< 0.001*
Self-distraction	0.14	0.009*	0.19	< 0.001*	0.22	< 0.001*	0.18	< 0.001*	0.19	< 0.001*	0.09	0.068
Denial	0.26	< 0.001*	0.20	< 0.001*	0.22	< 0.001*	0.15	0.004*	0.26	< 0.001*	0.29	< 0.001*
Venting	0.21	< 0.001*	0.18	0.001*	0.26	< 0.001*	0.16	0.003*	0.20	< 0.001*	0.19	< 0.001*
Substance use	0.04	0.381	0.05	0.275	0.02	0.630	−0.06	0.262	−0.04	0.407	0.13	0.014*
Behavioural disengagement	0.31	< 0.001*	0.32	< 0.001*	0.28	< 0.001*	0.22	< 0.001*	0.33	< 0.001*	0.36	< 0.001*
Self-blame	0.21	< 0.001*	0.16	0.002*	0.23	< 0.001*	0.19	< 0.001*	0.22	< 0.001*	0.16	0.002*

* *r*: Pearson’s correlation

* *P*-value < 0.05 shows significant
